# The Education of Mentally and Otherwise Defective Children

**Published:** 1903-08-15

**Authors:** 


### THE EDUCATION OF MENTALLY AND OTHERWISE DEFECTIVE CHILDREN.

IN 1899 the Education of Defective Children Act came into force, and the Liverpool School Board was the first, or one of the first, to adopt it and carry it out. Much progress has been made in that city, and there are now four schools entirely for children who are defective in some point or other. Attached to these schools are a band of specially-trained teachers, a nursing staff, and visiting medical officers. The scholars are not only collected and sent to the schools by the authorities, but in this case they are given a free dinner at the expense of the board. Each child goes to work with those similarly affected to himself, that is, the mentally deficient, the epileptics, and the cripples attend separate classes. The medical officer attached to the staff examines the children before they enter, and they all belong to that minority not compelled by law to receive the ordinary Board School education. Although boys as old as 16 are admitted, the teachers are, without exception, women, it having been demonstrated that in these departments women are more sympathetic, and hence more successful in obtaining good results than men. A great deal of "domestic" work has to be done for these children, and a large stock of patience is required.

The subjects taught vary considerably, as each defect has its peculiarities and its limitations, and all require that the classes should be small and the school hours short. In addition to the ordinary elementary teaching work has to be found for the hands of the scholars, and basket-making, carpentering, and other manual occupations supply the necessary incentives. Kindergarten work is also done in these schools, and where possible gardening, including the study of plant life, is among the subjects

It is satisfactory to know that the parents often appreciate what is being done for their children; and in even the most unpromising cases, such as the mentally defective, the improvement is often marked, and it is followed by a look of intelligence in the scholars which could hardly have been expected from their previous dull and apathetic state. The Liverpool School Board deserves much praise for so loyally and carefully carrying out the provisions of the Act, and they will get that satisfaction by noting the sharp contrast between the previous misery and degradation of the children and their present improved condition. Doubtless the example set by Liverpool will be widely followed, because it is the initiative that is so difficult in these things. To follow a good lead is easy enough.

